# Three-dimensional ERCP with cone-beam computed tomography: Novel approach to managing complex biliary strictures

**DOI:** 10.1055/a-2796-5509

**Published:** 2026-02-09

**Authors:** Kanika Garg, Gaurav Kakked, Thomas Wang, Christopher G Chapman, Ajaypal Singh, Irving Waxman, Neal A Mehta

**Affiliations:** 1 2468 Division of Digestive Diseases, Rush University Medical Center, Chicago, United States

**Keywords:** Pancreatobiliary (ERCP/PTCD), Strictures, ERC topics

## Abstract

**Background and study aims:**

Complex perihilar and intrahepatic biliary strictures present significant therapeutic challenges during endoscopic retrograde cholangiopancreatography (ERCP), with traditional two-dimensional fluoroscopy limiting spatial visualization. This prospective cohort study evaluated feasibility and clinical utility of novel three-dimensional (3D) fluoroscopic imaging utilizing cone beam computed tomography (CBCT) in ERCP for managing complex biliary strictures.

**Patients and methods:**

Twenty consecutive patients with complex biliary strictures underwent ERCP with CBCT at a single tertiary center between September 2023 and December 2024. All patients had previously failed selective cannulation attempts. Using a specialized fluoroscopic system, 360-degree rotational imaging was performed. Primary outcomes included technical success (selective cannulation rate) and clinical success (successful stenting without percutaneous drainage).

**Results:**

Stricture etiology was malignant in 60% and benign in 40%. CBCT achieved selective cannulation in 100% of cases and clinical success in 90%. Two patients required percutaneous drainage. 3D reconstruction influenced surgical planning in 10% of cases. Standard ERCP resulted in median entrance skin dose (ESD) of 156.0 mGy (range 67.3–273.0) and dose area product (DAP) of 44.4 (range 21.1–94.0] . The 3D reconstruction from CBCT contributed an additional median ESD of 174.0 mGy (range 137.0–240.0) and DAP of 53.1 Gy·cm ^2^ (range 41.8–73.8).

**Conclusions:**

ERCP with CBCT is technically feasible with high selective cannulation rates in complex biliary strictures. Although the technique requires additional radiation exposure, it may provide value in cases with difficult selective cannulation and surgical mapping for malignancies. Further studies are needed to define optimal patient selection and evaluate long-term outcomes.

## Introduction

Endoscopic retrograde cholangiopancreatography (ERCP) has evolved from a diagnostic tool to a primarily therapeutic procedure for a variety of pancreaticobiliary indications since its introduction in the 1960s 1 . Over the years, ERCP has become central to management of complex biliary pathology. Despite its widespread use, ERCP remains technically challenging in certain cases, particularly in management of perihilar or intrahepatic biliary strictures arising from conditions such as cholangiocarcinoma (CCA) or primary sclerosing cholangitis (PSC). These strictures present significant therapeutic difficulties, often requiring advanced techniques, judicious use of contrast, and careful procedure planning 2 .

Traditional ERCP relies on two-dimensional (2D) fluoroscopic imaging, which is limited in its ability to provide a comprehensive spatial understanding of complex biliary anatomy 3 . This limitation is particularly problematic when dealing with intrahepatic and perihilar strictures, where accurately localizing and selectively cannulating specific ducts can be arduous or even impossible using standard fluoroscopic guidance, because 2D imaging may fail to clearly delineate three-dimensional (3D) relationships despite passage of contrast 4 .

Cone beam computed tomography (CBCT) provides 3D imaging via 360-degree rotation of the x-ray source (the C-arm) and provides a promising adjunct to enhance ERCP capabilities in managing complex biliary strictures 5 . By providing a more comprehensive spatial representation of the biliary anatomy from multiple angles, CBCT has the potential to improve diagnostic accuracy, facilitate precise interventions, assist in future surgical planning, and potentially reduce need for alternative procedures such as percutaneous transhepatic biliary drainage (PTBD) 6 7 .

This prospective study aimed to assess feasibility, technical success, and clinical outcomes of CBCT-ERCP in diagnosis and treatment of perihilar and intrahepatic biliary strictures. We hypothesized that use of CBCT would enhance visualization, improve selective cannulation rates, and lead to more efficacious therapeutic interventions.

## Patients and methods

### Study design and patient selection

We conducted a prospective cohort study of consecutive patients with perihilar or intrahepatic strictures at a tertiary referral center between September 2023 and December 2024, evaluating CBCT-ERCP for relief of biliary obstruction. Adult patients scheduled for ERCP due to jaundice in the setting of these biliary strictures were considered for inclusion if they had previously failed selective cannulation of the target bile duct despite successful transpapillary access. Failed cannulation was defined as inability to successfully cannulate the target bile duct despite attempting multiple advanced techniques, which could include guidewire manipulation with varied wire types and angles, sphincterotome angulation and torque, patient positioning changes, and/or cholangioscopy guidance. Determination of failure was made at the discretion of the therapeutic endoscopist performing the procedure. Exclusion criteria included inability to provide informed consent, existing percutaneous biliary drainage, and unfeasible transpapillary biliary access based on prior endoscopic assessment due to duodenal/ampullary obstruction.

All patients underwent cross-sectional imaging with computed tomography (CT) with contrast or magnetic resonance cholangiopancreatography (MRCP) before the procedure for anatomical assessment and procedure planning. All procedures were performed by expert endoscopists with extensive experience in managing complex biliary strictures. The study was approved by the institutional review board (IRB) #23101204.

### Cone beam computed tomography

CBCT is an innovative imaging modality in which a radiation source emits slightly divergent x-rays for at least 180 degrees (typically ~200-degrees) in a circumferential manner around the target object to generate a 3D reconstructable dataset 8 9 . Although technically distinct from conventional CT, CBCT produces imagery remarkably similar to standard CT scans. However, in contrast to conventional CT, CBCT typically provides superior spatial resolution, albeit with somewhat noisier images. This characteristic makes CBCT particularly well-suited for imaging hard tissue (i.e. bones) and contrast-enhanced structures (i.e. biliary tree), and less optimal for soft tissue visualization 9 .

### Performance of CBCT-ERCP

Three-dimensional CBCT reconstruction of a patient's biliary system.Video 1

All patients with hilar strictures received prophylactic antibiotics prior to the procedure. Pre-procedure imaging was reviewed to determine target duct selection based on degree of dilatation, anatomical distribution of strictures, and clinical presentation.

CBCT-ERCP followed a standardized protocol. All procedures were performed with patients in the prone position under general anesthesia using a therapeutic duodenoscope (TJF-Q190, Olympus, Tokyo, Japan). Patients were positioned on a specialized fluoroscopic table with CBCT capabilities (Artis Q, Siemens Healthineers, Forchheim, Germany). Standard ERCP with selective wire-guided biliary cannulation was first attempted to target dilated intrahepatic ducts in an attempt to relieve the obstruction via stenting. If selective cannulation of the target duct with standard ERCP failed, CBCT was utilized. As patients were under general anesthesia with controlled ventilation; breath-holding was not required during CBCT image acquisition.

Standard accessories included either D.A.S.H. sphincterotome (Cook Medical, Bloomington, Indiana, United States) or an RX-39 sphincterotome (Boston Scientific, Marlborough, Massachusetts, United States), used with a 0.025-inch angled Visiglide guidewire (Olympus, Tokyo, Japan) for all cannulation attempts. When appropriate, balloon dilation was performed using either a Hurricane balloon dilator (Boston Scientific, Marlborough, Massachusetts, United States) or Soehendra biliary dilators (Cook Medical, Bloomington, Indiana, United States). Adjunctive cholangioscopy (SpyGlass system [Boston Scientific, Marlborough, Massachusetts, United States] or EyeMaxx system [Micro-Tech, Nanjing, China]) was employed selectively when direct visualization was necessary for optimal wire placement or when CBCT guidance alone was insufficient. Direct single-operator cholangioscopy (DSOC) also was utilized at the discretion of the endoscopist.

**Fig. 1:**
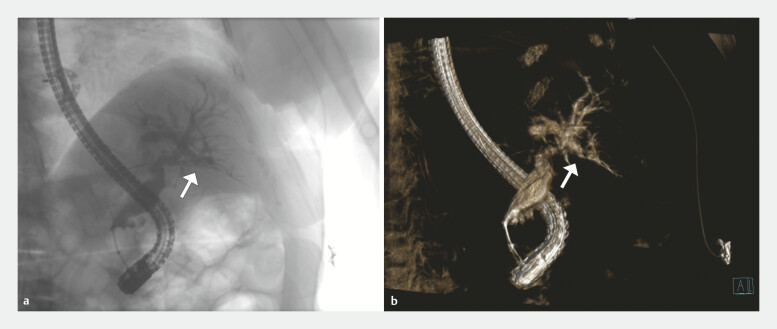
CBCT imaging of the biliary system with white arrows indicating the target bile duct. **a** Two-dimensional rotational cholangiogram showing contrast-filled biliary ducts. **b** Three-dimensional CBCT reconstruction of the same biliary system.

High-volume contrast injections were administered with a standard extraction balloon inflated in the distal bile duct. CBCT image acquisition was then performed using the dynaDR acquisition protocol, which provides visualization of the biliary anatomy while minimizing radiation exposure to the patient ( Fig. 1 ). This protocol performs a 200-degree rotation over 6 seconds, capturing multiple images (0.50 degree per frame) of the biliary anatomy. This generates an immediate 2D rotational cholangiogram, followed shortly by the completion of the 3D reconstruction ( Video 1 ). Subsequent selective cannulation was guided by the enhanced 3D anatomical understanding provided by the CBCT images in conjunction with overlayed 2D standard fluoroscopy. Two radiation metrics were recorded: entrance skin dose (ESD), which measures radiation dose at the point where the x-ray beam enters the patient’s skin (expressed in milligray, mGy) and dose area product (DAP), which represents total radiation energy delivered to the patient by considering both dose and irradiated area (expressed in gray·square centimeter, Gy·cm²).

### Data collection and analysis

Comprehensive data were collected for each procedure, including patient demographics, procedure details, radiation exposure, and clinical outcomes. Radiation exposure was measured separately for standard ERCP and 3D reconstruction phases. Technical success was defined as successful selective cannulation of the target duct. Clinical success was defined as ability to relieve biliary obstruction with endoscopic stenting without requiring PTBD.

### Statistical analysis

Statistical analyses were performed using R version 4.1.0 (R Foundation for Statistical Computing, Vienna, Austria). Continuous variables are presented as median (interquartile range) based on distribution skewness. Categorical variables are expressed as frequencies and percentages.

## Results

### Patient and procedure characteristics

Twenty patients (median age 68.5 years, 45% male) underwent CBCT-ERCP ( Table 1 ) after failure of standard ERCP. Biliary stricture etiology was malignant in 60% (12/20) and benign in 40% (8/20). Among malignant strictures, six were cholangiocarcinoma and six were metastatic disease. Among benign strictures, three were postsurgical, two were PSC, two were due to intrahepatic stones, and one was immunoglobulin G4-related cholangiopathy. Anatomic distribution of hilar strictures according to Bismuth classification was Bismuth II in eight patients (40%), Bismuth IIIa in one patient (5%), and Bismuth IV in 11 patients (55%). Median total procedure time was 62.1 minutes (58.4–71.2).

**Table:** **Table 1** Patient characteristics and stricture details.

Characteristic	Median [IQR] or N (percentage)
Demographics	
Age (years)	68.5 [63.0–73.0]
Male	9 (45)
Stricture etiology	
Malignant	12 (60)
Cholangiocarcinoma	6 (30)
Metastatic disease	6 (30)
Benign	8 (40)
Postsurgical	3 (15)
Primary sclerosing cholangitis	2 (10)
Intrahepatic stones	2 (10)
IgG4-related cholangiopathy	1 (5)
Bismuth classification	
Bismuth II	8 (40)
Bismuth IIIa	1 (5)
Bismuth IV	11 (55)
IQR, interquartile range.	

### Clinical outcomes

CBCT aided selective wire cannulation in 100% of cases (20/20) ( Table 2 ). Clinical success was achieved in 90% of cases (18/20). Two patients (10%) required PTBD despite successful selective wire cannulation in the target duct: one due to worsening peripheral liver metastases and one due to complete right anterior duct obstruction from aberrant surgical clips following cholecystectomy. In 10% of cases (2/20), CBCT reconstructions played a role in surgical planning.

**Table:** **Table 2** Procedure and clinical outcomes with radiation exposure data.

Outcome	Median [IQR] or N (percentage)
Procedure outcomes	
Selective cannulation rate	20 (100)
Clinical success rate	18 (90)
Radiation exposure	
Standard ERCP ESD	156.0 mGy [67.3–273.0]
Standard ERCP DAP	44.4 Gy·cm ^2^ [21.1–94.0]
CBCT ESD	174.0 mGy [137.0–240.0]
CBCT DAP	53.1 Gy·cm ^2^ [41.8–73.8]
Clinical outcomes	
Required PTBD	2 (10)
Influenced surgical planning	2 (10)
CBCT, cone beam computed tomography; DAP, dose area product; ESD, entrance skin dose; PTBD, percutaneous transhepatic biliary drainage.	

Of note, DSOC was employed in three patients (15%) before CBCT and in five patients (25%) during the CBCT procedure for direct visualization and wire guidance. Among the 15 patients (75%) in whom CBCT was used without concurrent cholangioscopy during the procedure, selective cannulation success was achieved in 100% of cases (15/15), with clinical success in 93% (14/15). In those patients where cholangioscopy was employed during the CBCT procedure, CBCT provided the 3D roadmap for target duct identification whereas cholangioscopy offered complementary direct visualization for wire manipulation.

One patient (5%) developed post-procedure cholangitis and required percutaneous transhepatic biliary drainage (one of the two patients requiring PTBD as described above). No cases of pancreatitis, bleeding, or perforation occurred.

### Radiation exposure data

For standard ERCP, median ESD was 156.0 mGy (67.3–273.0), with a median DAP of 44.4 Gy·cm ^2^ (21.1–94.0). CBCT contributed an additional median ESD of 174.0 mGy (137.0–240.0) and DAP of 53.1 Gy·cm ^2^ (41.8–73.8). Median total fluoroscopy time for the entire CBCT-ERCP procedure was 17.9 minutes (13.9–21.5).

## Discussion

### Clinical impact

This study demonstrates feasibility and clinical utility of CBCT-ERCP in management of complex biliary strictures when conventional ERCP fails. The high rate of successful selective cannulation (100%) and clinical success (90%) are particularly noteworthy given the complexity of cases included in the study.

ERCP with stenting in the setting of perihilar and intrahepatic duct obstruction are among the most time-consuming and difficult advanced therapeutic procedures. Selective duct cannulation requires tactful manipulation of the sphincterotome, wire, and endoscope, frequently requiring multiple attempts to achieve cannulation. By providing enhanced spatial orientation, CBCT allows for more precise ductal navigation and may reduce the number of attempts required for successful cannulation 10 11 . This improved anatomical visualization can also facilitate internal drainage of ducts that may have otherwise required percutaneous or higher risk endoscopic ultrasound-guided approaches 12 .

CBCT served as the primary adjunctive imaging modality, with DSOC reserved for cases requiring direct visualization —highlighting a practical, stepwise approach when standard ERCP maneuvers fail. Utilization of multiple complementary techniques in select patients demonstrates the comprehensive approach taken when standard ERCP techniques with or without DSOC failed. In cases in which both modalities were used, CBCT provided spatial orientation and target duct identification, whereas cholangioscopy offered direct luminal visualization for wire guidance. These technologies served distinct but synergistic roles—CBCT answered where to navigate, whereas cholangioscopy facilitated how to advance the wire.

CBCT represents an advancement in spatial visualization capabilities that address specific needs in managing complex biliary strictures through enhanced 3D anatomical understanding. Although CBCT directly influenced surgical planning in only a subset of patients, its utility in delineating segmental ductal involvement may be particularly valuable in preoperative mapping of perihilar cholangiocarcinoma 13 . Detailed 3D reconstructions can provide valuable information about tumor involvement, status of segmental ducts, and potential resection margins.

PTBD is commonly pursued when endoscopic drainage fails but is associated with higher complication rates, patient discomfort, and need for multiple interventions 6 14 15 16 . By improving selective cannulation success, CBCT-ERCP could significantly reduce reliance on PTBD, improving outcomes, patient comfort, and resource utilization 17 . In our cohort, two patients required PTBD after ERCP and selective cannulation. These cases reflect limitations of endoscopic drainage in general (peripheral metastatic disease and complete surgical clip obstruction) rather than failures of the CBCT-ERCP technique itself.

### Safety profile

Radiation exposure associated with CBCT requires careful consideration in clinical practice. Compared with standard ERCP, CBCT contributed an additional median DAP of 53.1 Gy·cm² in our study. Published diagnostic reference levels from a large multicenter study demonstrate that ERCP for proximal malignant biliary obstruction results in higher radiation exposure (median ESD 118 mGy, median DAP 27 Gy·cm²) compared with general ERCP procedures (median ESD 69 mGy, median DAP of 16 Gy·cm²) 18 . Combined ERCP plus CBCT exposure in our study (total median ESD 330 mGy, total median DAP 97.5 Gy·cm²) exceeds these benchmarks. This likely reflects our select patient population of complex cases that had previously failed standard ERCP, representing more challenging anatomic and technical scenarios. However, the CBCT addition (53.1 Gy·cm²) represents a relatively modest proportional increase compared with the initial ERCP component and may be justified in cases in which it avoids need for PTBD.

Current consensus among U.S. public health and radiation safety agencies supports a linear, no-threshold model for radiation-induced cancer risk, meaning any added exposure may incrementally increase lifetime risk 19 . This risk must be weighed against the clinical context, particularly given that many of these patients already have malignancy and may have limited alternative options.

In our study, we utilized the dynaDR acquisition protocol, which is specifically designed to provide adequate visualization of contrast-filled structures while minimizing radiation exposure to the patient. These findings are consistent with previously published data on the CBCT-ERCP experience in Europe, which demonstrated higher radiation doses with CBCT compared with conventional ERCP in most cases, although there were still instances in which standard ERCP cases utilized a higher radiation dose than CBCT 20 .

When ERCP fails, the primary alternative is PTBD, which involves significantly higher radiation exposure than standard ERCP 21 . Studies of interventional radiation-guided PTBD procedures report mean DAPs of 70 Gy·cm² or higher per procedure for biliary drainage 22 23 . Therefore, additional radiation from CBCT may be justified if it successfully avoids PTBD in a significant proportion of patients.

Moreover, because this technology is in its early stages, there is potential for optimizing CBCT acquisition protocols to reduce radiation exposure further 20 24 . Adherence to ALARA (As Low As Reasonably Achievable) principles through careful patient selection, use of optimized protocols, and procedure planning can help minimize unnecessary exposure 25 . We recommend judicious use of CBCT for cases most likely to benefit from selective duct cannulation. Utilizing CBCT in this manner could help balance the risk/benefit profile of this technology. Ultimately, if CBCT reduces need for repeated ERCP attempts or avoids PTBD, it may lead to a reduction in cumulative radiation exposure for some patients over their treatment course.

### Limitations

These results should be interpreted within the context of some limitations that affect generalizability. First, this single-center study was conducted at a tertiary referral center by experienced therapeutic endoscopists. This level of expertise may not be representative of all clinical settings and the technical success rates observed may not be reproducible at centers with different operator experience or case volumes. Lack of randomization introduces potential selection bias because all procedures were performed by highly experienced operators who may have had inherent advantages in achieving successful outcomes regardless of imaging modality used.

Second, the patient population represents a highly selected cohort of complex cases that had already failed previous ERCP attempt. Although this selection criterion strengthens the clinical relevance of our findings—demonstrating utility in genuinely challenging cases—it also limits broader applicability. Centers with different referral patterns or case complexity may experience different success rates with this technique.

Third, the learning curve associated with CBCT-ERCP utilization was not formally assessed in this study. Optimal use of 3D reconstruction requires both technical proficiency in image acquisition and interpretive skills for anatomical analysis. The number of cases required to achieve competency with this technique remains undefined and may vary significantly among endoscopists.

Fourth, an important limitation of CBCT utility is the requirement for contrast opacification of the biliary tree. In cases in which upstream ducts cannot be opacified—often due to lobar atrophy or complete obstruction—CBCT cannot provide additional guidance. Importantly, such non-opacified segments typically should not be targeted for drainage given risk of introducing infection into obstructed, non-drainable ducts. Therefore, CBCT is appropriately limited to cases in which target ducts can be opacified with contrast, which aligns with sound clinical practice of selectively draining accessible biliary segments.

In addition, the definition of failed cannulation was based on endoscopist judgment rather than standardized time or attempt thresholds, which may introduce variability in patient selection.

Finally, due to the small sample size evaluating this novel technology, validation of the results in larger cohort and randomized controlled studies (RCTs) is necessary. However, fluoroscopic units are not routinely replaced due to a variety of factors, including cost. Because this technology is novel and not widely available on existing fluoroscopic units, multicenter evaluations would be difficult. In addition, this study focused on immediate technical success and short-term clinical improvements, leaving long-term outcomes as an area for further investigation.

### Implementation considerations

The role of CBCT should be considered within the broader context of advanced biliary imaging. Digital cholangioscopy emerged over 20 years ago as a powerful tool for managing complex biliary pathology, offering direct visualization of the biliary epithelium and targeted tissue sampling 26 . Although cholangioscopy excels in characterizing mucosal abnormalities and guiding biopsies, CBCT can be utilized synergistically by providing superior spatial understanding of the biliary architecture, particularly for high-grade hilar and intrahepatic strictures. The complementary nature of these technologies suggests potential value in their combined use for complex cases, although cost implications of implementing both systems require careful consideration.

Introduction of CBCT-ERCP necessitates specific training considerations as well. In our experience, proficiency in obtaining images and interpreting CBCT reconstructions required approximately five cases. Key components of the learning curve include proper patient positioning for image acquisition and interpretation of CBCT reconstructions from various angles.

### Cost implications

In the current economic climate, hospitals continue to push for cost-effectiveness and minimizing disposables. Purchase and implementation of CBCT-capable systems warrant thoughtful evaluation of these economic factors. The initial capital investment for CBCT-capable fluoroscopy systems ranges significantly, typically between $500,000 to $1 million, with additional maintenance and training costs. However, this investment should be weighed against potential cost savings from reduced need for repeat procedures, fewer failed ERCPs requiring subsequent PTBD, and decreased length of hospital stay. In our cohort, the 100% selective cannulation success rate suggests potential cost benefits through avoiding additional procedures. A formal cost-effectiveness analysis comparing CBCT-ERCP with conventional ERCP followed by PTBD in cases of failure would be valuable for healthcare systems considering implementing this technology.

### Future directions

These results should be validated in multicenter studies across institutions with varying levels of endoscopic expertise to better define generalizability of CBCT-ERCP. Future research evaluating this technology should be able to address these limitations and explore additional aspects of CBCT-ERCP. Multicenter RCTs comparing CBCT-ERCP with conventional ERCP in complex biliary strictures would be beneficial. Long-term follow-up studies are needed to assess impact on patient outcomes, including overall survival data in the setting of malignancy and stricture recurrence rates for benign etiologies. Prospective studies comparing CBCT-ERCP with PTBD for technical success, clinical outcomes, patient satisfaction, and cumulative radiation exposure would be valuable. For wider implementation of this technology, investigating the learning curve associated with its use and development of standardized protocols would be important. Ultimately, comprehensive cost-effectiveness analyses are necessary to evaluate economic implications of implementing CBCT systems in ERCP suites.

## Conclusions

In conclusion, this study demonstrates that CBCT-ERCP is a feasible, safe, and effective technique for managing complex biliary strictures. The technology's ability to enhance visualization, improve selective cannulation rates, and potentially reduce need for percutaneous procedures is promising for advancing the field of advanced biliary imaging. Increased radiation exposure and cost considerations require incorporation of its use in specific treatment paradigms when traditional ERCP fails. As we continue to evaluate CBCT-ERCP with larger-scale studies, there is promise that it could emerge as a useful tool for managing challenging biliary strictures.
